# Close Relationship between West Nile Virus from Turkey and Lineage 1 Strain from Central African Republic

**DOI:** 10.3201/eid2102.141135

**Published:** 2015-02

**Authors:** Koray Ergunay, Tamas Bakonyi, Norbert Nowotny, Aykut Ozkul

**Affiliations:** Hacettepe University, Ankara, Turkey (K. Ergunay);; University of Veterinary Medicine Vienna, Vienna, Austria (T. Bakonyi, N. Nowotny);; Szent István University, Budapest, Hungary (T. Bakonyi);; Sultan Qaboos University, Muscat, Oman (N. Nowotny);; Ankara University, Ankara (A. Ozkul)

**Keywords:** West Nile virus, WNV, lineage 1, flavivirus, complete genome, Turkey, viruses, Central African Republic, strains, sequence identity, *Suggested citation for this article*: Ergunay K, Bakonyi T, Nowotny N, Ozkul A. Close relationship between West Nile virus from Turkey and lineage 1 strain from Central African Republic. Emerg Infect Dis [Internet]. 2015 Feb [*date cited*]. http://dx.doi.org/10.3201/eid2102.141135

## Abstract

We sequenced West Nile viruses (WNVs) from Turkey and found close relationships to WNV lineage 1 strain ArB310/67 from the Central African Republic, distinct from other WNVs circulating in the Mediterranean Basin, eastern Europe, and the Middle East. These findings suggest independent introductions of WNV strains from Africa to the Middle East.

West Nile virus (WNV; family *Flaviviridae*, genus *Flavivirus*) (*1*) is a mosquito-borne avian virus that can cause febrile illness and potentially life-threatening neuroinvasive disease in mammalian hosts, predominantly in humans and horses (*2*). WNV is widely distributed in Africa; the Middle East; central, southern and eastern Europe; southwestern Russia; southwestern Asia and Australia (*3*); and North, Central, and South America (*4*,*5*).

Initial evidence for WNV circulation in Turkey was provided by serosurveillance reports (*6*). Since 2009, sporadic human and equine cases and limited disease outbreaks have demonstrated the occurrence of symptomatic infections comprising self-limiting febrile diseases as well as central nervous system infections resulting in occasional deaths (*6*–*8*). Current data reveal widespread WNV circulation in Turkey, including virus detection in competent mosquito species (*8*–*10*). To determine the lineage and relationships of WNVs circulating in Turkey, we analyzed the complete genome sequence of the initially reported WNV isolate from Turkey and several partial sequences from isolates detected from various sources and locations in this country during 2011–2013.

## The Study

WNV strain T2 was isolated from a horse originating from Eskisehir Province (39°24′N–31°02′E), Central Anatolia, Turkey; the horse had febrile illness beginning on January 18, 2011, and neurologic signs subsequently developed (*8*). The complete genome sequence was amplified by using continuous reverse transcription PCR assays and WNV lineage 1–specific primers as previously described (*11*). Amplification products were directly sequenced in both directions (Microsynth, Balgach, Switzerland) using the amplification primers. The resulting overlapping partial sequences were verified by BLAST search (http://www.ncbi.nlm.nih.gov/blast/) and compiled to 1 continuous sequence. Three regions, not covered by the reactions, were amplified with novel forward (f) and reverse (r) primers: 4275f, 5′-GACTATCGCGGGGCTCATGT-3′; 4686r, 5′-GCCCGCTCCTGCTTGATAAC-3′; 4940f, 5′-GGGCCGTGACTTTGGACTTC-3′; 5290r, 5′-TGGGCAGTCCTCTCAGTGCT-3′; 7968f, 5′-TGAAGAGCCCCAACTAGTGC-3′; and 8277r, 5′-CCGTGAGAGTGGGTTTCTGA-3′. (Region numbers refer to nucleotide positions in WNV strain HNY1999, GenBank accession no. AF202541). These regions were then sequenced, and the sequences were compiled with the previously determined sequences. The complete genome of WNV strain T2 was compared with genomes of 32 other WNV strains, and phylogenetic analysis was performed. For sequence handling and phylogenetic analyses, we used CLC Main Workbench version 5.5 (CLCBio, Aarhus, Denmark) and MEGA version 6.0.5 (http://www.megasoftware.net/).

**Figure 1:**
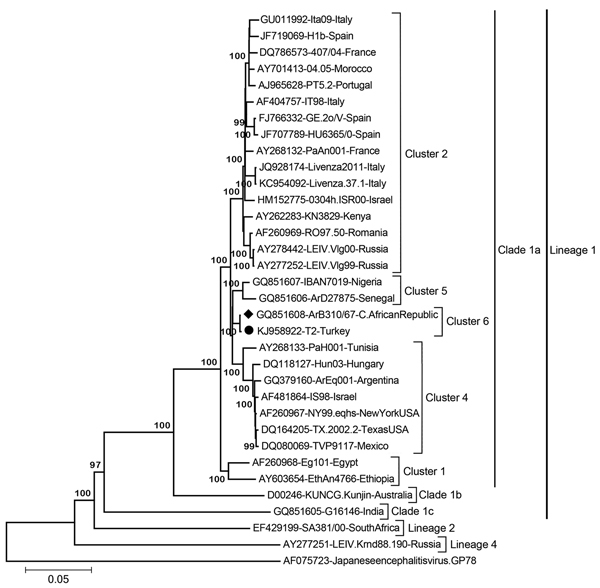
Neighbor-joining phylogenetic tree constructed on the basis of the complete genomic nucleotide sequences of selected West Nile virus (WNV) strains, including isolate T2 from Turkey (black dot) and the closely related strain ArB310/67 from the Central African Republic (black diamond). Major WNV lineages, clades, and clusters are indicated, except for the local cluster 3 (*12*). Bootstrap values of major branches are given for 1,000 replicates. Virus strains are described by GenBank accession number, name, and country of isolation. Japanese encephalitis virus strain GP78 served as outgroup. Scale bar indicates substitutions per site.

The T2 genome consists of 11,026 nt (GenBank accession no. KJ958922); the 5′ and 3′ untranslated regions comprise 93 (nt 1–93) and 630 (nt 10396–11026) bases, respectively. The putative open reading frame (nt 93–10395) was translated to a 3,433-aa polypeptide that encodes viral capsid (C, positions 1–105); C-anchor peptide (ER, positions 106–123); premembrane protein (prM, positions 124–290); envelope protein (E, positions 291–791); and nonstructural (NS) proteins NS1 (positions 792–1143), NS2A (positions 1144–1374), NS2B (positions 1375–1505), NS3 (positions 1506–2124), NS4A (positions 2125–2250), peptide 2k (positions 2251–2273), NS4B (positions 2274–2528), and NS5 (positions 2529–3433). Comparison of the complete nucleotide sequence with those of several other lineage 1 WNV strains revealed 0.7% (strain ArB310/67) to 20.6% (strain G16146, clade 1c) divergence (data not shown). Phylogenetic analyses of the nucleotide and amino acid sequences demonstrated, supported by high bootstrap values, that strain T2 clusters within WNV lineage 1 clade 1a viruses, forming a distinct cluster together with WNV strain ArB310/67, which was isolated in 1967 in the Central African Republic (Figure 1). Similar or identical topologies were observed in maximum-likelihood and UPGMA trees (data not shown).

**Figure 2:**
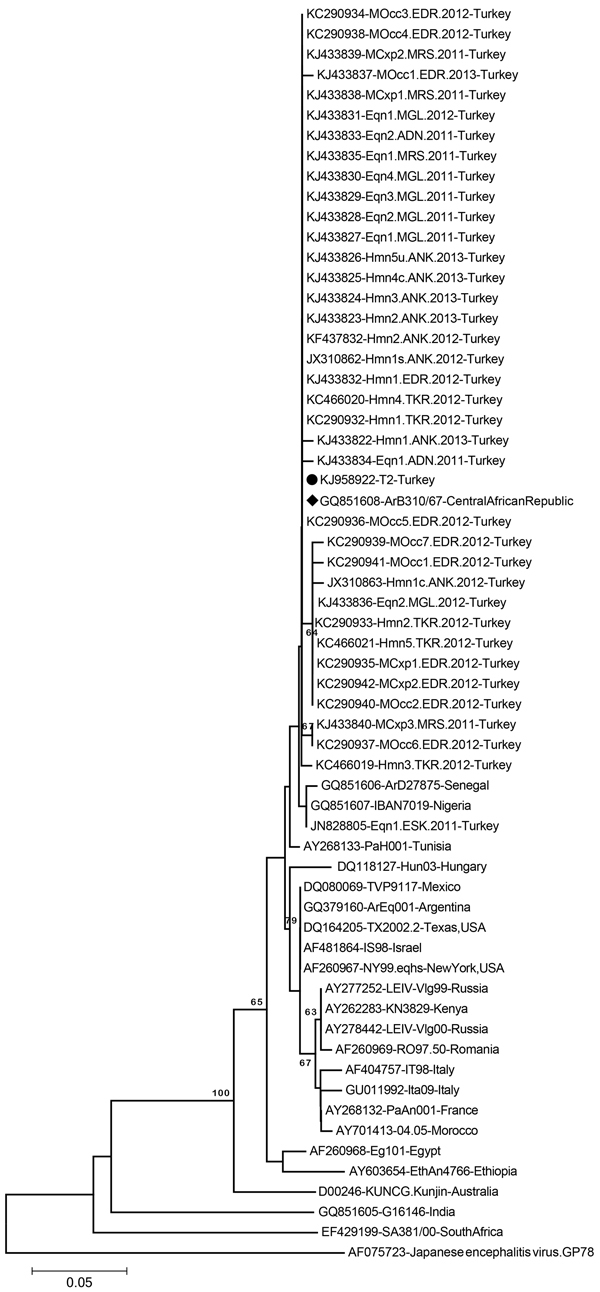
Neighbor-joining phylogenetic tree constructed on the basis of the West Nile virus (WNV) partial envelope gene nucleotide sequences (183 nt) of 38 WNV strains from Turkey and selected global strains. Black dot indicates WNV isolate T2 from Turkey, and black diamond indicates the closely related strain ArB310/67 from the Central African Republic. Bootstrap values of major branches are given for 1,000 replicates. GenBank accession number, name, and country of isolation are given for global strains. Local sequences are indicated by GenBank accession number, host, location, and year. ADN, Adana Province; ANK, Ankara Province; Cxp, *Culex pipiens*; EDR, Edirne Province; Eqn, equine; ESK, Eskisehir Province; Hmn, human; M, mosquito, MGL, Mugla Province; MRS, Mersin Province; Occ, *Ochleratatus caspius*; TKR, Tekirdag Province. Scale bar indicates substitutions per site.

To evaluate whether T2 is the main WNV strain circulating in Turkey or whether divergent strains are co-circulating, we investigated another 37 WNV isolates, detected during 2011–2013 from different geographic regions of Turkey and from human, equine, and mosquito samples. From these samples, only partial envelope (E) gene sequences were available; these sequences consisted of 212–256 nt and corresponded to amino acid positions 174–255 in the T2/NY99 WNV polyprotein and have been deposited in GenBank (accession nos. JN828805, JX310862, JX310863, KC290932–KC290942, KC466019–KC466021, KF437832, and KJ433822–KJ433840). These 37 partial E gene sequences exhibited 98.8%–100% nucleotide identities to each other, regardless of collection date, geographic area, and source of isolation (data not shown). Neighbor-joining phylogenetic analysis of a common 183-nt stretch of these isolates revealed that all but 1 clustered together with the T2 and ArB310/67 strains (Figure 2). Maximum-likelihood and UPGMA analyses yielded similar results (data not shown). One isolate (GenBank accession no. JN828805), identified from a horse in February 2011 in Central Anatolia, clustered in a sister subclade with 2 other older WNV strains from Africa (strain IBAN7019, isolated in 1965 in Nigeria, and strain ArD27875, isolated in 1979 in Senegal).

WNV lineage 1 strains have a widespread geographic distribution throughout Africa, Europe, the Middle East, and North America, and have been divided into 3 clades, of which clade 1a contains the largest number of strains; these strains can be further grouped into 6 distinct clusters (*12*). The phylogenetic analyses in our study confirm that the various clusters are well separated and that the T2 strain and 36 of 37 other WNV strains from Turkey are located in cluster 6, together with strain ArB310/67 from the Central African Republic (Figures 1, 2). However, subclustering of the sequences in cluster 6 (Figure 2) is statistically poorly supported because of the relatively short sequences analyzed and the few common nucleotide substitutions.

Amino acid substitutions that delineate WNV strains belonging to cluster 1 (NS4B-S11N), cluster 3 (NS2A-A224T), cluster 4 (E-T126I, NS4A-V85A), and the eastern European subtype of cluster 2 (NS1-L206F, NS2B-A103V, NS3-T249P and NS5-T898I) were not found in the T2 WNV strain from Turkey (*12*). Likewise, substitutions that have become established in the North America WNV populations (E-K291R, NS4A-A85T, and NS5-K318R) were absent from this strain. However, the NS1-A70S substitution noted in cluster 2 viruses was observed as A70V in strain T2. Furthermore, NS3-P249T and NS5-V258A substitutions, reported in several strains of the Mediterranean subtype (*12*), were detected in the T2 strain. These findings suggest that the T2 strain evolved independently from other Mediterranean strains from a common ancestor of African origin.

The T2 strain retained the original WNV envelope protein glycosylation motif (E154–156, NYS) and lacked substitutions associated with neurovirulence attenuation (NS4b-C102S, NS2A-A30P) and increased virulence for crows and humans (NS3-T249P) (*12*,*13*). However, the E-V159A change in the WN02 genotype that was associated with a shorter extrinsic incubation period in *Culex* spp. mosquitoes (*14*) was noted as V159F in this isolate. A few other amino acid variations with unknown implications were also observed (data not shown).

## Conclusions

The evaluation of the complete nucleotide and amino acid sequences of the T2 WNV isolate from Turkey revealed a close genetic relationship with strain ArB310/67, isolated in 1967 in the Central African Republic. These 2 virus strains form a distinct genetic cluster within WNV lineage 1a strains. This observation is supported by analysis of partial E gene sequences detected in temporally and spatially separated sources in Turkey. The investigated circulating WNV strains from Turkey proved to be genetically distinct from viruses circulating in eastern Europe, the Mediterranean region, and the Middle East. However, several amino acid substitutions detected in viruses from throughout the Mediterranean region were identified in strain T2, including those that might affect virus replication in vector mosquitoes. A recent report on a lineage 2 WNV obtained from a human patient in Iran in 2009 also revealed 99% nucleotide identity to a WNV strain collected in the Central African Republic (*15*). These findings suggest independent introductions of WNV strains of African origin to the Middle East, potentially by migratory birds, and the accumulation of adaptive changes during circulation.
